# GPR115 Contributes to Lung Adenocarcinoma Metastasis Associated With LAMC2 and Predicts a Poor Prognosis

**DOI:** 10.3389/fonc.2020.577530

**Published:** 2020-11-20

**Authors:** Yingjing Wang, Muqi Shi, Nan Yang, Xiaoyu Zhou, Liqin Xu

**Affiliations:** ^1^ Department of Clinical Biobank, Affiliated Hospital of Nantong University, Nantong, China; ^2^ Department of Pathology, Medical School of Nantong University, Nantong, China; ^3^ Department of Clinical Medicine, Medical School of Nantong University, Nantong, China; ^4^ Department of Respiratory and Critical Care Medicine, Affiliated Hospital of Nantong University, Nantong, China; ^5^ Department of Respiratory Medicine, Affiliated Hospital of Nantong University, Nantong, China

**Keywords:** GPR115, non-small cell lung cancer, lung adenocarcinoma, prognosis, metastasis

## Abstract

GPR115, a member of the adhesion G protein-coupled receptor family, is dysregulated in many cancers. However, the expression and function of GRP115 in non-small cell lung cancer (NSCLC) is not clear. Here, we examined the expression pattern, clinical significance, and function of GPR115 in NSCLC by analysis of clinical specimens and human cell lines and bioinformatics analysis. Immunohistochemical analysis of clinical samples showed that GPR115 was significantly upregulated in NSCLC tissues compares with normal lung epithelial tissue (P < 0.05). And GPR115 overexpression is an independent prognostic factor for 5-year overall survival of NSCLC patients [hazard ratio (HR)=1.625, P = 0.008]. Interestingly, higher expression of GPR115 was strongly correlation with differentiation level (P = 0.027), tumor size (P = 0.010), lymph node metastasis (P = 0.022), tumor-node-metastasis stage (P = 0.008), and poor prognosis of lung adenocarcinoma (LUAD, all P = 0.039), but not lung squamous cell carcinoma (LUSC, P > 0.05). Moreover, downregulation of GPR115 by RNA interference in human lung cancer lines inhibited cell proliferation, migration, and invasion. Preliminary bioinformatic analysis confirmed that GPR115 was closely associated with LAMC2 (Spearman correlation coefficient=0.67, P < 0.05), which was accumulated in ECM-receptor interaction and focal adhesion. Consistent with these findings, deceased of GPR115 was associated with E-cadherin, N-cadherin and Vimentin confirmed by western blot. In conclusion, these data suggest that GPR115 may play a role in the tumor growth and metastasis and may have utility as a diagnostic and prognostic marker for LUAD, but not LUSC.

## Introduction

Lung cancer is responsible for 18.4% of all deaths from cancer and is thus the leading cause of cancer-related death worldwide. Non-small cell lung cancer (NSCLC) accounts for 80–85% of total lung cancer cases (1, 2). The most common histological subtypes of NSCLC are lung adenocarcinoma (LUAD) and lung squamous cell carcinoma (LUSC) which account for approximately 50% and 30% of NSCLC cases, respectively (3). Many tumor-associated genes are known to be aberrantly expressed in NSCLC (4–6) but its pathogenesis is still unclear. Even with the advent of targeted therapy and immunotherapy, the 5-year survival rate from NSCLC is only 16% (7). Thus, further understanding of the molecular mechanisms underlying the development and progression of NSCLC is needed to provide new directions for developing more effective diagnostic and therapeutic approaches.

G protein-coupled receptors (GPCRs) are characterized by seven transmembrane α-helices and are the largest family of membrane receptors in many species. GPCRs play crucial roles in the regulation of many biological functions (8, 9), and unsurprisingly, dysregulation of their expression and/or activity is associated with numerous diseases. Recent data suggest that GPCRs are involved in the initiation and progression of cancer, and they have been shown to regulate cancer cell proliferation, invasion, metastasis, migration, and adhesion, as well as angiogenesis (10). GPR115 (G protein-coupled receptor 115) is a member of the adhesion GPCR (aGPCR) family, which consists of membrane-bound receptors with a long N-terminus (11). However, the current research on GPR115 is very limited. Some researchers found that GPR115 plays an important role in the pathogenesis of inflammatory skin diseases, and may be related to the treatment of glucocorticoids in these diseases (12). The latest study showed that Gpr115 plays an important role in the development of ectodermal organs, and may regulate enamel mineralization by regulating the expression of Car6 in ameloblasts (13). Moreover, some researchers have found that GPR115 is upregulated in breast cancer, colon adenocarcinoma, and thyroid carcinoma, and its methylation is closely related to the treatment of lung cancer (14, 15). They suggested that GPR115 may have the potential to promote tumor development, but the specific molecular mechanism is still unclear. It is worth noting that we found that GPR115 mRNA is elevated in NSCLC and is closely related to patient survival. In order to verify the effect of GPR115 on the occurrence and development of NSCLC, we analyzed GRP115 levels in NSCLC clinical specimens and cell lines to determine its expression pattern, clinical significance, and biological effects. We also performed bioinformatic analysis to identify candidate genes with expression related to that of GPR115 that may be involved in its function.

## Materials and Methods

### Human Tissue Specimens and Clinical Information

A total of 393 formalin-fixed paraffin-embedded tissue samples collected from the Department of Clinical Bio-bank, Affiliated Hospital of Nantong University between 2005 and 2011. Of the 393 samples, 298 were NSCLC (156 LUAD, 99 LUSC, and 43 lung adenosquamous carcinoma (LUAS)) and 95 were matched paracancerous normal tissues. The clinical characteristics were obtained from the patients’ medical records and included complete clinical information and follow-up records. None of the patients had been treated with radiotherapy, chemotherapy, or targeted drugs before surgery, and none had a history of cardiovascular or other neoplastic diseases. A tissue microarray of the samples was fabricated using a manual Tissue Microarrayer System Quick Ray (UT06, UNITMA, Korea) as previously described (16). This study was approved by the Human Research Ethics Committee of the Affiliated Hospital of Nantong University, Jiangsu, China.

### Immunohistochemistry Analysis (IHC)

Tissue microarray sections were deparaffinized and rehydrated through a graded series of alcohol (100%, 95%, and 75%). Antigens were retrieved by immersing the sections in citrate buffer at 99°C for 30 min, and endogenous peroxidase activity was then quenched by incubation in 3% H_2_O_2_. GPR115 expression was detected by incubating the sections with a 1:200 dilution of anti-GPR115 antibody (1:200 dilution, HPA007158, Atlas Antibodies) at 4°C overnight followed by horseradish peroxidase-conjugated secondary antibody at room temperature for 1 h. Color development was achieved with a 3,3′-diaminobenzidine solution (Kit-0015, Maxim Biotechnologies, Fuzhou, China) and the sections were then counterstained with hematoxylin. Slides were scanned using an Automated Quantitative Pathology Imaging System (PerkinElmer, Shanghai, China).

Staining was scored by a pathologist using a semi-quantitative system based on the staining intensity and percentage of cells stained. Intensity was scored as: 0 (negative, blue), 1+ (weak, yellow), 2+ (positive, brown-red), and 3+ (strong, brown). The H-score was calculated from the sum of the four staining intensity scores × the percentage of cells staining at that intensity, which gave a final score between 0 and 300. The median score, 183, was selected as the cutoff value for dichotomizing samples into two group with low (0–183) and high (184–300) GPR115 expression.

### Cell Lines and Cell Culture

The human LUAD cell lines A549, H1975, SPCA, and H1650 were obtained from the Cell Bank, Type Culture Collection, Chinese Academy of Science (Shanghai, China) and were maintained in RPMI 1640 medium (Thermo Scientific/Gibco, USA) supplemented with 10% fetal bovine serum (Thermo Scientific/Gibco) in a humidified atmosphere of 5% CO_2_ at 37°C.

### Western Blot Analysis

Cells were lysed in 500 μl of high-efficiency RIPA buffer (R0010, Solarbio, Beijing, China) containing Phenylmethanesulfonyl fluoride (PMSF, P0100, Solarbio, Beijing, China) on ice for 2 h and then centrifuged for 10 min at 12,000 rpm. Protein concentrations in the supernatants were measured using the BCA method (BL521A, Biosharp, Hefei, Anhui, China). Proteins were resolved by 10% SDS-PAGE and transferred to polyvinylidene fluoride membranes (IPVH00010, Millipore, Massachusetts, USA). After blocking with 5% nonfat dried milk, the membranes were incubated at 4°C overnight with primary antibodies specific for: GPR115 (1:1,000, HPA007158, Atlas Antibodies, Sweden), E-cadherin (1:100, ab76055, Abcam, Cambridge, UK), N-cadherin (1:1,000, ab18203, Abcam, Cambridge, UK), Vimentin (1:100, ab8978, Abcam, Cambridge, UK), and glyceraldehyde 3-phosphate dehydrogenase (GAPDH, 1:2000, AB-M-M001, Goodhere, Hangzhou, China). The membranes were then incubated with corresponding secondary antibodies and visualized using Enhanced Chemiluminescence reagent (Beyotime Biotechnology, Shanghai, China). Images were captured using a chemiluminescence imager (ChemiScopr 5300 Pro) and analyzed using ImageJ 1.51j8 software (National Institutes of Health, Bethesda, MD, USA).

### RNA Interference

Three small interfering RNAs (siRNAs) were designed to specifically target GPR115: hs-GRP115-si-1 sense: 5′-GAUCCAAGAUUCACCUAAA-3′; hs-GRP115-si-2 sense: 5′-GGAUUUACAUGUAAUCAAA-3′; hs-GRP115-si-3 sense: 5′-CAUUGAGAGUGUAGCUCAA-3′ (Biomics Biotech, Nantong, China). And A control scrambled siRNA sequence was also synthesized (sense: 5’-UUCUCCGAACGUGUCACGU-3’). The optimal conditions for transient transfection into NSCLC cell lines, determined in preliminary experiments, were 75 pmol/well siRNA and 7.5 μl/well Lipofectamine 3000 (HanBio, Shanghai, China).

### Cell Proliferation Assay

SPCA and H1650 cell proliferation was measured using a Cell Counting Kit (CCK8, C2581, Sigma Aldrich, St. Louis, MO, USA) according to the manufacturer’s description. In brief, the cells were cultured in RPMI 1640 medium supplemented with 10% FBS for 0, 24, 48, or 72 h and then 10 μl of CCK8 reagent was added to each well. Cells were cultured in the dark for an additional 4 h, and the absorbance at 450 nm was measured using a multifunctional enzyme labeling instrument (MK3, Thermo Fisher Scientific). The experimental group was repeated three times, and the final data obtained was statistically and analyzed by Graphpad Prism 8.0 software. And one-way ANOVA was used to analysis the relationship between GPR115 expression and the proliferation ability of LUAD cells.

### Wounding Healing Assay

After the cells grew to 90% confluence, the wound was scraped off in the monolayer using a micropipette tip and placed in RPMI 1640 medium containing 1% FBS. The non-adherent cells were washed gently with PBS. The wells were visualized immediately before (0 h) and then at 24 and 48 h after incubation at 37°C using an inverted microscope (DMI3000B, Leica). The experimental group was repeated three times, and the wounding healing rate was quantified by Image J 1.51j8 software (National Institutes of Health, Bethesda, MD, USA). Finally, the association between GPR115 expression and cell metastasis was analyzed by two-way ANOVA by Graphpad prism v8.0 software.

### Transwell Invasion Assay

Transwell chambers (Costar; Corning, New York, NY, USA) coated with Matrigel (BD Biosciences, Franklin Lakes, USA) were used to measure the invasion of SPCA and H1650 cells. Cells were harvested at 24 h after transfection, resuspended in medium, and added to each upper chamber at 10^5^ cells/well. 600 µl RPMI-1640 that contained 10% FBS was added to the lower chambers. Plates were incubated for 48 h, and the upper chamber was removed, washed twice with phosphate buffered saline (PBS; Maxim Biotechnologies, Fuzhou, China), fixed with pre-cooled 4% paraformaldehyde for 30 min, and stained with crystal violet for 10 min. The total number of invaded cells on the lower side of the membrane were visualized and counted under an inverted microscope at 200× magnification (DMI3000B, Leica). Each experimental group was repeated three times, and the invaded cell numbers were counted by Image J 1.51j8 software (National Institutes of Health, Bethesda, MD, USA). Two-way ANOVA was used to analysis the relationship between GPR115 expression and cell invasion by Graphpad prism v8.0 software.

### Statistical Analysis

All data were analyzed using Prism 8 (GraphPad, La, Jolla, CA, USA) or SPSS V.20.0 (IBM, Armonk, NY, USA) software. The relationship between GPR115 expression level and clinicopathological features was assessed using Pearson’s χ^2^ test and Cox regression analysis. The Kaplan–Meier method was used to evaluate the relationship between gene expression and 5-year survival. P < 0.05 was considered statistically significant.

### Bioinformatic Analysis

The mRNA microarray and clinicopathology information in LUAD and LUSC were directly obtained by The Cancer Genome Atlas (TCGA, https://portal.gdc.cancer.gov/). One-way ANOVA was used to analysis the relationship among GPR115 expression, stage and lymph node metastasis by Graphpad prism v8.0 software. And the limma package in R (Version 4.0) was applied to normalization and screen the differential expression genes (DEGs) between high GPR115 expression and low GPR115 expression. The cutoff criteria were FDR-p value < 0.05 and |logFC| > 1. Then WGCNA (Version 4.0), which was discover gene modules that were highly related with clinical information (17), was used to explore the co-expression similarity network of DEGs, and the power value was set when scale free R^2^ > 0.9. Next, the GO and KEGG analysis were constructed by ClusterrProfiler package (Version 4.0) to identify the significant signaling pathway of module DEGs, and P < 0.05 was thought to be statistically significant. The heatmap of DEGs based differential expression of GPR115 was performed by pheatmap package. Finally, the Spearman’s and Pearson correlation analysis was obtained by cBioPortal database (http://www.cbioportal.org) (18).

## Results

### GPR115 Expression Is Upregulated in NSCLC Tissues and Predicts Poor Prognosis

**Figure 1:**
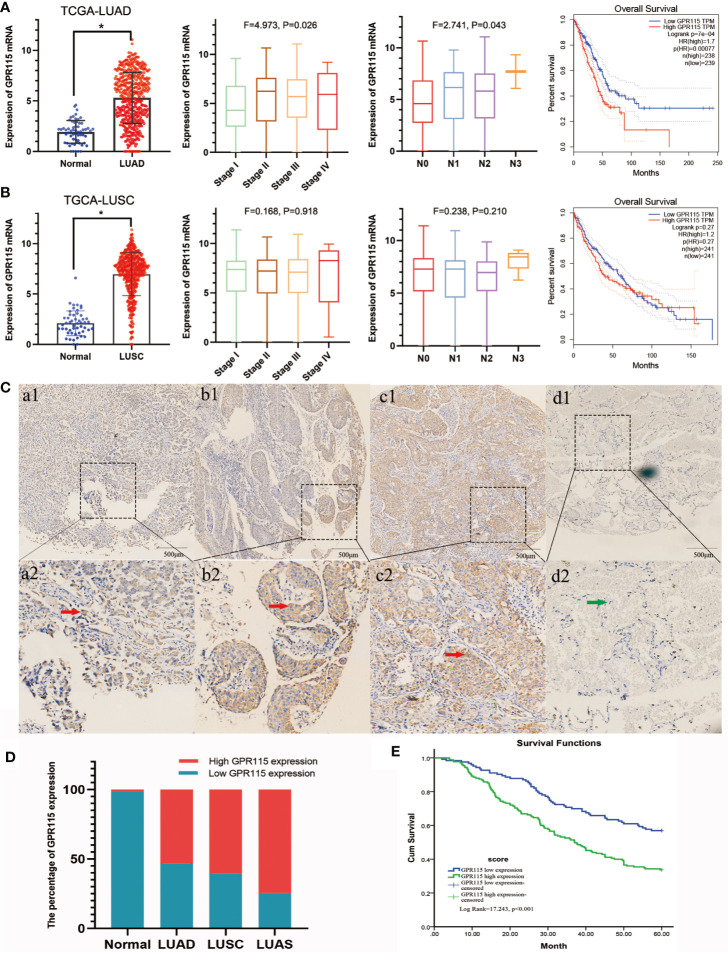
Expression of GPR115 mRNA and protein in NSCLC. The GPR115 mRNA expression and the relationship between tumor stage, lymph node metastasis and overall survival in TCGA-LUAD **(A)** and TCGA-LUSC **(B)**. **(C)** Positive for GPR115 protein expression in LUAD tissues (a1-a2), LUSC tissues (b1-b2) and lung adenosquamous carcinoma tissues (c1-c2). Negative expression of GPR115 in normal lung epithelial tissue (d1-d2). a1-d1 had a bar with 500 μm; a2-d2 were a partial enlarge picture of a1-d1. GPR115 protein expression on cancerous cell cytoplasm was indicated by red arrows, and negative GPR115 protein expression on non-cancerous cells was indicated by green arrows. **(D)** The relationship of GPR115 expression and tumor type. **(E)** The Kaplan-Meier overall survival curves of GPR115 in NSCLC. Blue line, GPR115 low expression; green line, GPR115 high expression. *P < 0.05 indicated significant difference.

Firstly, we found the highly expression of GPR115 in LUAD and LUSC tissues by TCGA analysis. The expression of GPR115 was significance different with LUAD stage and lymphatic metastasis (P < 0.05). In addition, the higher GPR115 expression, the worse the overall survival of LUAD patients, but not of LUSC (**Figures 1A, B**).

Subsequently, IHC analysis of 393 clinical specimens showed that GPR115 was present in both the cytoplasm and membrane of cancer cells (**Figure 1C**). Expression of GRP115 was quantified using a combination of staining intensity score and the percentage of positively stained cells (score range 0–300). Using the median staining score of 183 as a cutoff, we classified the samples as having low (<183) or high (≥184) expression of GRP115. Using this system, more than half of the NSCLC tumor specimens were classified as having high GRP115 expression (58.72%, 175/298) compared with control samples (0.01%, 1/95, χ^2^ = 115.1, P < 0.001, **Figure 1D**). Correlation analysis (Pearson’s χ^2^) indicated that high GPR115 expression was significantly (P ≤ 0.05) associated with tumor type, differentiation, tumor size, lymph node metastasis, and tumor-node-metastasis (TNM) stage in NSCLC patients (χ^2^ = 6.475, 18.473, 7.664, 17.269 and 13.816, respectively; all P < 0.05, **Table 1**). And GPR115 expression level, gender, differentiation, TNM stage, tumor size, and lymph node metastasis were significantly correlated with 5-year survival of NSCLC patients in univariate analysis (hazard ratio [HR]=1.967, 0.655, 1.458, 1.642, 1.429, and 1.580, respectively, all P < 0.05). But in multivariate analysis, only GPR115 expression level remained potential independent prognostic factors (HR = 1.625, P = 0.008, **Table 2**). Kaplan–Meier survival curve analysis confirmed that the enhanced of GPR115 was associated with poor prognosis in NSCLC patients (**Figure 1E**).

**Table 1:** Correlation of GPR115 expression in NSCLC tumor tissues with Clinicopathology characteristics.

Characteristic	Low expression	High expression	Pearson χ2	P
**Total**	123(41.28%)	175(58.72%)		
**Gender**			1.149	0.284
** Male**	76(39.09%)	120(60.91%)		
** Female**	46(45.54%)	55(54.46%)		
**Age**			6.475	0.039*
**≤60**	73(46.79%)	83(53.21%)		
**>60**	39(39.39%)	60(60.61%)		
**Smoking\**			0.021	0.884
** No smoking**	67(42.95%)	89(58.05%)		
** Smoking**	23(41.82%)	32(58.18%)		
** Unknown**	33	45		
**Type**			6.475	0.039*
**Lung** **adenocarcinoma**	73(46.79%)	83(53.21%)		
**Lung squamous** **cell carcinoma**	39(39.39%)	60(60.61%)		
**Adenosquamous** **carcinoma**	11(25.58%)	32(74.42%)		
** Unknown**	0	13		
**Differentiation**			18.473	<0.001*
**Well**	15(50.00%)	15(47.37%)		
**Middle**	88(49.16%)	96(50.84%)		
**Poor**	14(22.00%)	42(80.00%)		
**Unknow**	6	11		
**T**			7.664	0.022*
**1**	57(50.89%)	55(49.11%)		
**2**	57(35.85%)	102(64.15%)		
** 3 and 4**	7(29.17%)	17(70.83%)		
** Unknown**	2	1		
**N**			17.269	<0.001*
**0**	89(50.68%)	86(49.14%)		
**1**	17(25.76%)	49(74.24%)		
**2**	15(27.78%)	39(72.22%)		
**Unknown**	2	1		
**M**			0.149	0.699
**M0**	119(41.18%)	170(58.82%)		
** M1**	2(33.33%)	4(66.67%)		
**Unknown**	2	1		
**TNM stage**			13.816	0.001*
**IA and IB**	63(53.85%)	24(46.15%)		
**IIA and IIB**	39(34.82%)	41(65.18%)		
**IIIA and IIIB and** **IV**	19(28.79%)	18(71.21%)		
** Unknown**	2	1		

*P < 0.05.

**Table 2:** Univariate and multivariable analysis of prognostic factors for 5-year survival in NSCLC.

	Univariate analysis			Multivariate analysis (adjusted for age)		
	HR	P >|z|	95% CI	HR	P >|z|	95% CI
**GPR115** ** High vs. Low**	1.967	<0.001*	1.420–2.725	1.625	0.008*	1.135–2.328
**Age (years)** **≤60 vs >60**	1.112	0.504	0.815–1.517			
**Gender** ** Male vs. Female**	0.655	0.013*	0.469–0.916	0.709	0.064	0.493–1.020
**Smoking** ** No vs. Yes**	1.336	0.156	0.896–1.993			
**Type** ** 1 vs. 2 vs. 3**	1.108	0.331	0.901–1.363			
**Differentiation** ** Well vs. Middle vs. Poor**	1.458	0.007*	1.110–1.916	1.270	0.118	0.941–1.713
**TNM stage** ** 1 vs. 2 vs. 3**	1.642	<0.001*	1.355–1.990	1.435	0.090	0.946–2.177
**T** ** 1 vs. 2 vs. 3**	1.429	0.005*	1.114–1.834	1.003	0.984	0.733–1.372
**N** ** 0 vs. 1 vs. 2**	1.580	<0.001*	1.322–1.887	1.181	0.364	0.824–1.692
**M** ** M0 vs. M1**	0.992	0.988	0.316–3.107			

*P < 0.05.

Type 1, Lung adenocarcinoma; Type 2, lung squamous cell carcinoma; Type 3, Lung adenosquamous carcinoma.

TNM 1, IA and IB; TNM 2, IIA and IIB; TNM 3, IIIA; IIIB and IV.

### GPR115 Expression Is Associated With Clinicopathological Features, Including Prognosis in LUAD, But Not in LUSC

**Figure 2:**
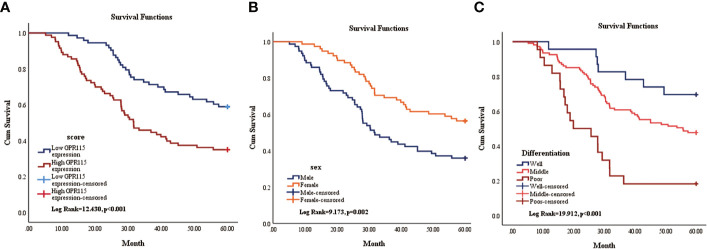
Kaplan-Meier survival curve of overall survival of LUAD patients. **(A)** The relationship of GPR115 expression and overall survival. Blue line indicated low GPR115 expression. Red line indicated high GPR115 expression. **(B)** The relationship between sex and OS of LUAD patients. Orange line indicated female and blue line meant male. **(C)** The relationship of differentiation and OS. Blue line, orange line and red line indicated well, middle and poor differentiation, respectively.

Then the subgroup analysis of the relationship between tumor GPR115 expression and clinicopathological features in the 156 LUAD and 98 LUSC cases identified several significant correlations; namely, between GRP115 expression in LUAD samples and differentiation, tumor size, lymph node infiltration and TNM staging (χ^2^ = 7.241, 9.299, 7.642 and 9.702, respectively; all P < 0.05) and between GPR115 expression in LUSC samples and differentiation (χ^2^ = 9.240, P = 0.010, **Table 3**). For LUAD patients, univariate analysis identified GPR115 expression, gender, smoking, degree of differentiation, TNM stage, tumor size, and lymph node metastasis were significantly related to 5-year overall survival (all P < 0.05). In multivariate analysis, high GPR115 expression, gender and degree of differentiation remained independent prognostic risk factors (HR = 1.706, 0.485 and 1.914, respectively; all P < 0.05). For LUSC patients, only TNM stage and lymph node metastasis were significantly associated with survival in univariate analysis, but neither were significant in multivariate analysis (**Table 4**). Kaplan–Meier curves for the LUAD patients were shown in **Figure 2**.

**Table 3:** Correlation of GPR115 expression in tumorous tissues with clinicopathologic characteristics in LUAD and LUSC patients.

Characteristic	LUAD				LUSC			
	Low expression	High expression	Pearson χ2	P	Low expression	High expression	Pearson χ2	P
**Total**	73(46.79%)	83(53.21%)			39(39.39%)	60(60.61%)		
**Gender**			0.026	0.873			3.238	0.072
** Male**	36(46.15%)	42(53.85%)			34(36.96%)	57(63.04%)		
** Female**	37(47.44%)	41(52.56%)			5(71.42%)	2(28.57%)		
**Age**			0.058	0.080			0.125	0.723
** ≤60**	35(50.00%)	35(50.00%)			11(41.31%)	15(57.69%)		
** >60**	38(44.19%)	48(55.81%)			28(38.36%)	45(61.64%)		
** Smoking**			2.867	0.090			2.502	0.114
** No smoking**	47(49.47%)	48(50.53%)			14(35.90%)	25(64.10%)		
** Smoking**	5(27.78%)	13(72.22%)			14(56.00%)	11(44.00%)		
** Unknown**	18	25			11	24		
**Differentiation**			7.241	0.027*			9.240	0.010*
**Well**	14(60.87%)	9(39.13%)			1(14.29%)	6(85.71%)		
**Middle**	53(49.53%)	54(50.47%)			30(50.85%)	29(49.15%)		
**Poor**	5(22.73%)	17(77.27%)			7(21.88%)	25(78.13%)		
**Unknow**	5	5			1	0		
**T**			9.299	0.010*			0.800	0.670
**1**	46(58.97%)	32(41.03%)			8(32.00%)	17(68.00%)		
**2**	25(34.72%)	47(65.28%)			23(39.66%)	35(60.34%)		
** 3 and 4**	2(33.33%)	4(66.67%)			6(41.67%)	7(58.33%0		
** Unknown**	0	0			2	1		
**N**			7.642	0.022*			5.245	0.073
**0**	55(55.00%)	45(45.00%)			26(47.27%)	29(53.85%)		
**1**	10(30.30%)	23(69.70%)			7(35.00%)	13(65.00%)		
**2**	8(34.78%)	15(65.22%)			4(19.05%)	17(80.95%)		
**Unknown**	0	0			2	1		
**M**			0.321	0.571			0.634	0.426
**M0**	71(46.71%)	81(53.29%)			37(38.95%)	58(61.05%)		
** M1**	2(50.00%)	2(50.00%)			0(0.00%)	1(100.00%)		
**Unknown**	0	0			2	1		
**TNM stage**			9.702	0.008*			3.691	0.158
**1**	39(61.90%)	24(38.10%)			17(45.95%)	20(54.05%)		
**2**	24(36.92%)	41(63.08%)			14(42.42%)	19(57.58%)		
**3**	10(35.71%)	18(64.29%)			6(23.08%)	20(76.92%)		
**Unknown**	0	0			2	1		

*P < 0.05.

TNM 1, IA and IB; TNM 2, IIA and IIB; TNM 3, IIIA, IIIB and IV.

**Table 4:** Univariate and multivariable analysis of prognostic factors for 5-year survival in LUAD and LUSC.

	LUAD						LUSC					
	Univariate analysis			Multivariate analysis (adjusted for age)			Univariate analysis			Multivariate analysis		
	HR	P >|z|	95% CI	HR	P >|z|	95% CI	HR	P >|z|	95% CI	HR	P >|z|	95% CI
**GPR115** ** High vs. Low**	2.192	0.001*	1.401–3.428	1.706	0.039*	0.940–3.097	1.735	0.193	0.833–2.442			
**Age (years)** ** ≤60 vs. >60**	1.223	0.364	0.792–1.887				0.818	0.477	0.471–1.422			
**Gender** ** 1 vs. 2**	0.516	0.003*	0.333–0.798	0.485	0.028*	0.254–0.926	0.902	0.842	0.327–2.489			
**Smoking** ** No vs. Yes**	2.517	0.003*	1.378–4.595	1.615	0.201	0.755–3.367	0.804	0.531	0.407–1.589			
**Differentiation** ** 1 vs. 2 vs. 3**	2.410	<0.001*	1.578–3.681	1.914	0.026*	1.081–3.391	0.850	0.482	0.540–1.337			
**TNM stage** ** 1 vs. 2 vs. 3**	1.814	<0.001*	1.374–2.395	1.018	0.967	0.439–2.357	1.823	<0.001*	1.312–2.531	1.481	0.167	0.848–2.585
**T** ** 1 vs. 2 vs. 3**	1.910	<0.001*	1.340–2.722	1.212	0.542	0.653–2.247	0.964	0.871	0.621–1.497			
**N** ** 0 vs. 1 vs. 2**	1.709	<0.001*	1.331–2.195	1.715	0.107	0.889–3.309	1.744	<0.001*	1.287–2.363	1.278	0.354	0.761–2.144
**M** ** M0 vs. M1**	1.137	0.857	0.028–4.626				4.229	0.159	0.567–31.521			

*P < 0.05.

Gender 1, Male, Gender 2, Female.

Differentiation 1, well; Differentiation 2, Middle; Differentiation 3, Poor.

TNM 1, IA and IB; TNM 2, IIA and IIB; TNM 3, IIIA, IIIB, and IV.

### Downregulated of GPR115 Blocked Cell Proliferation

**Figure 3:**
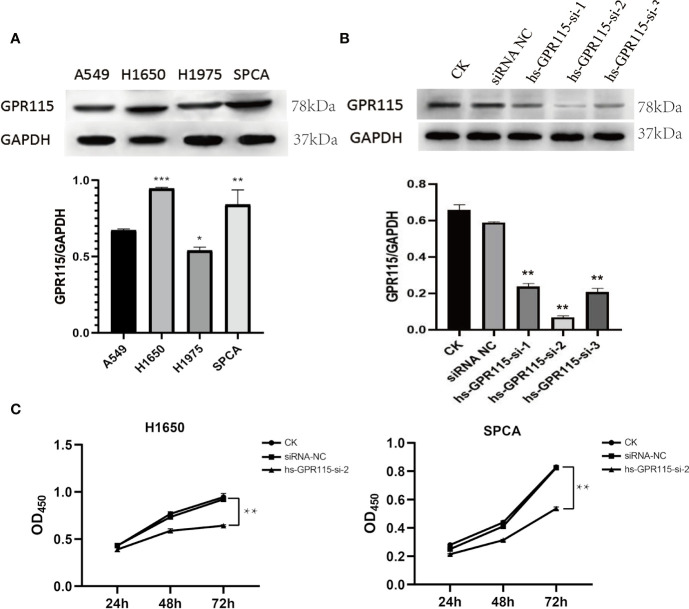
Expression of GPR115 in LUAD cell lines and Effect of GPR115 on proliferation of LUAD cells. **(A)** The relative protein expression of GPR115 in four LUAD cell lines. **(B)** The western blot analysis to select the most efficient siRNA in siGPR115-transfected cells, hs-GPR115-si-2 has the highest inhibition efficiency. **(C)** CCK8 experiments demonstrated that the proliferation of LUAD cells was affected by the intervention of GPR115 expression. Data are presented as means ±SD, * mean that P < 0.05, ** mean that P < 0.01, *** mean that P < 0.001.

Due to the remarkable tumor promotion effect of GPR115 in LUAD, we analyzed the expression of GPR115 protein in four human LUAD cell lines (H1650, H1975, A549, and SPCA) by western blotting. Based on their higher GRP115 expression level, H1650 and SPCA lines were selected for subsequent experiments (**Figure 3A**). To investigate the function of GPR115, the cells were transfected with one of three siRNAs (hs-GPR115-si-1, -2, and -3) or a control sequence (siRNA-NC). As shown in **Figure 3B**, the GRP115-specific siRNAs significantly decreased GPR115 expression compared with siRNA-NC (P < 0.05). The most efficient knockdown was observed with hs-GPR115-si-2.

Next, we evaluated the effect of knockdown of GPR115 on the proliferation of H1650 and SPCA cells by CCK8 experiment. The results suggested that GPR115 knockdown significantly suppressed the proliferation of H1650 and SPCA cells, and the efficiency increased with time (**Figure 3C**).

### Knock Down of GPR115 Inhibited Lung Cancer Migration and Invasion

**Figure 4:**
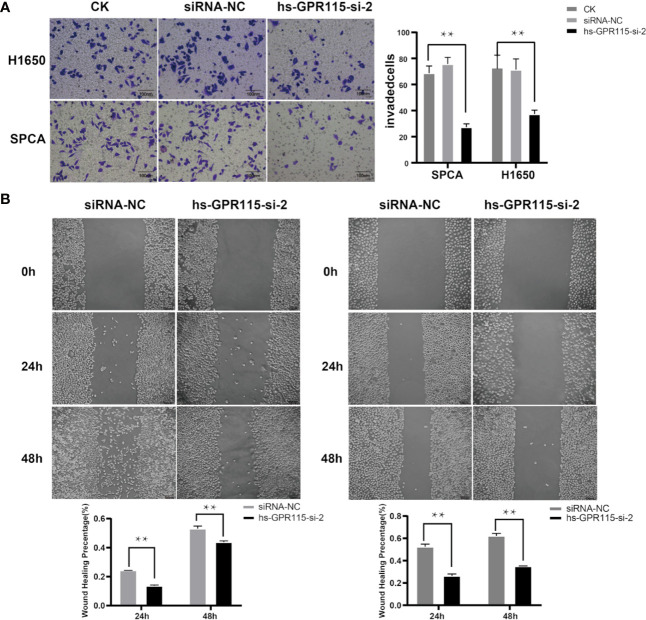
Effect of GPR115 on migration and invasion of LUAD cells. **(A)** Silencing GPR115 by hs-GPR115-si-2 reduced cell invasion by Transwell assay in H1650 and SPCA cell lines, respectively (Typical photomicrographs are presented). **(B)** Down-regulated of GPR115 inhibited the migration of LUAD cell lines by wound-healing assay. The scale bar means 100 μm. Data are presented as means ±SD, ** P < 0.01.

Cancer metastasis is the main reason for tumor recurrence and high mortality, and more than 90% of cancer-related death occur in metastatic tumor rather than primary tumors (19). To examine the effect of GRP115 in metastasis-related phenotype in LUAD, we first measured the cell migration by wound healing assay, which showed that the migration ability of H1650 and SPCA was significantly suppressed compared with NC cells (**Figure 4A**). We also assessed the invasion properties of LUAD cells by Matrigel Transwell assay. Similar with the effect of GPR115 regulation on CCK8, silencing of GPR115 significantly inhibited H1650 and SPCA cell invasion ability (**Figure 4B**).

### Screening of Differentially Expression Genes Associated GPR115

**Figure 5:**
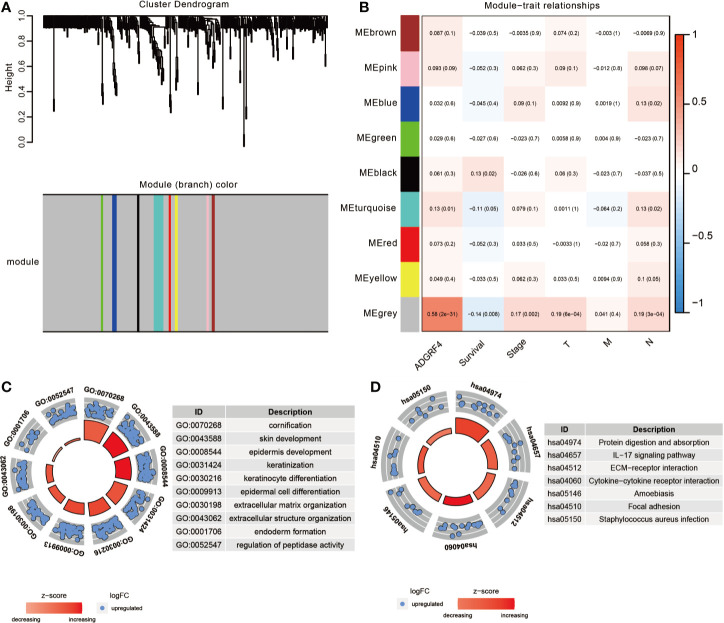
The function enrichment analysis of DEGs. **(A)** The WGCNA dendrogram for 378 up-regulated DEGs. **(B)** Heatmap of the correlation between co-expression module eigengenes and clinical traits (including GPR115 expression group). Each cell contained the corresponding correlation coefficient and p-value. The GO biological function analysis **(C)** and KEGG pathway analysis **(D)** of DEGs in gray module.

To detect genes that may be related to GPR115, we screened for genes with expression significantly correlated with that of GRP115 (P <0.05 and logFC>1) in TCGA-LUAD cohort, using limma package. A total of 908 genes were identified as significantly related with high expression of GPR115, containing 378 upregulated genes and 530 downregulated genes. Then the gene co-expression modules of up-regulated genes were constructed by WGCNA package (The downregulated genes were not correlated with overall survival of LUAD patients). By limiting the power value to 6, a total of 9 co-expression modules were obtained, and the gray module showed significantly associated with GPR115 expression, overall survival, tumor stage, tumor size and lymph node metastasis (all P < 0.05, **Figures 5A, B**).

### Identification of GPR115-Related Signaling Pathway

To identify the significant signaling pathway, the GO and KEGG enrichment analysis of gray module genes were performed. GO analysis revealed significant enrichment in cornification, skin, epidermis cell and keratinization development, extracellular matrix organization and so on (**Figure 5C**). In KEGG analysis, the genes were mainly enriched in “Protein digestion and absorption”, “IL-17 signaling pathway”, “ECM-receptor interaction”, “Cytokine-cytokine receptor interaction” and “Focal adhesion”, which suggested that GPR115 might be an intermediary for epithelial cell growth and metastasis (**Figure 5D**).

### The Molecular Mechanism of GPR115 Regulating Tumor Metastasis

**Figure 6:**
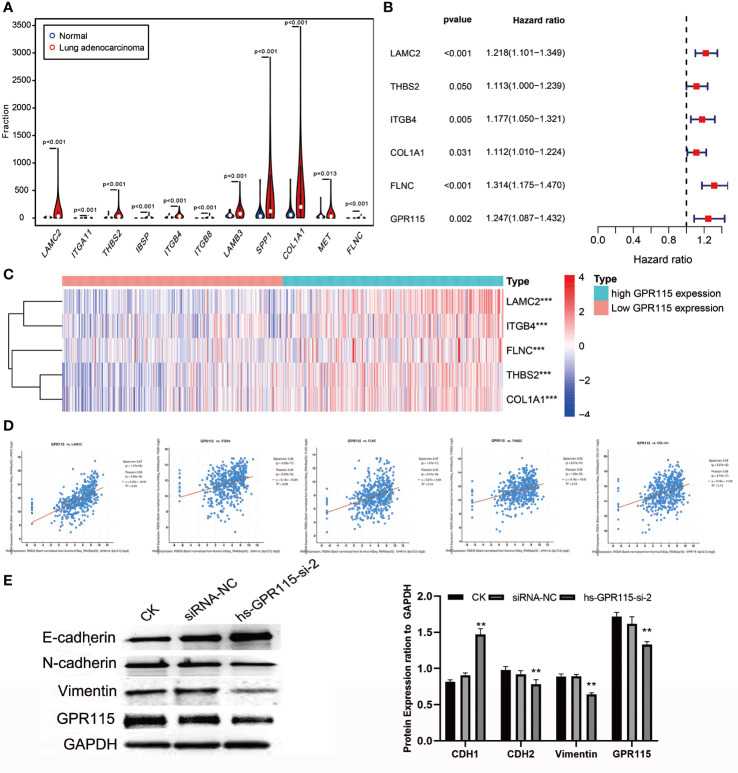
The molecular mechanism of GPR115 regulating tumor metastasis. **(A)** The differential expression of ECM receptor interaction/Focal adhesion related genes in LUAD. **(B)** The relationship of metastasis-related genes with overall survival of LUAD patients. **(C)** Heatmap showed the relationship of GPR115 expression level and corresponding survival-related genes. **(D)** Scatter plot showed correlation of GPR115 and LACM2, ITGB4, FLNC, THBS2 and COL1A1. P < 0.05 was considered statistically significant. **(E)** Western Blot confirmed that GPR115 promotes the downregulated of E-cadherin and the elevated of N-cadherin and Vimentin, **P < 0.01.

As the significantly enrichment signaling pathway, ECM-receptor interaction and Focal adhesion played an important role in tumor proliferation, adhesion and metastasis in multiply cancers. The violin plot showed the metastasis-related genes were higher expression in LUAD tumor tissues (**Figure 6A**), but only LAMC2, THBS2, ITGB4, COL1A1 and FLNC showed positively correlated with poorer survival of LUAD (**Figure 6B**) and the expression level of GPR115 (**Figure 6C**). Furthermore, Cbioportal software was applied to identified the Spearman’s and Person correlation coefficient between GPR115 and 5 differential expression genes, and the results pointed that LACM2 was most relevant to GPR115 (Spearman r=0.67, Pearson r=0.58, P < 0.05, **Figure 6D**).

LAMC2 is an epithelial-specific basement membrane protein that plays an important role in promoting tumor EMT. For EMT transformation, the mesenchymal proteins containing N-cadherin and Vimentin are enhanced and epithelial protein- E-cadherin is inhibited. Consistent with this possibility, we found that siRNA-mediated silencing of GRP115 in the lung cancer cell lines resulted in upregulation of E-cadherin, an epithelial cell marker, and downregulation of the mesenchymal cell markers N-cadherin and Vimentin (**Figure 6E**).

## Discussion

In this study, we first confirmed that GPR115 was significantly overexpression in NSCLC tissues than normal lung epithelial tissue, which consistent with the higher expression found in breast cancer and colon cancer (14). And higher GPR115 level was closely associated with tumor type, differentiation, tumor size, lymph node metastasis and TNM staging of NSCLC. And higher GPR115 expression was established as independent risk factors for NSCLC. But after further analysis by pathology type, we found that high GPR115 protein expression was significantly associated with the tumor progression and prognosis of LUAD, but not LUSC. The prognostic value reveled by this study was inconsistent with the results of Weimin Zhang et al. (12). This may be due to the fact that they used TRIM58 as the main variable to screen GPR115 as a differential gene, and then analyzed the significance of the prognostic risk by multiple genes risk scoring system in LUSC, which lacking the rigor of univariate. In summary, our research indicated that GPR115 might be a potential link between tumor progression and prognosis survival, which may serve as a biomarker for LUAD treatment.

aGPCRs are evolutionarily conserved and play crucial roles in physiological functions such as immunity, fertility, and neuronal function; however, they are also known to be involved in the development of various tumors (20–23). Most aGPCRs contain numerous domains associated with cell–cell and cell–matrix interactions, including the epidermal growth factor-like, thrombospondin, leucine-rich, and immunoglobulin domains, and cadherin repeats, and there is increasing evidence for the involvement of some aGPCRs in regulating cell proliferation and metastasis (11). For example, mice lacking GPR56 develop brain malformations due to aberrant migration of nerve cells (24). ADGFR5 (CD97) induces RhoA activation by interacting with the lysophosphatidic acid receptor 1 in thyroid cancer (25). Similar with other aGPCRs, we further demonstrated that knockdown of GPR115 expression in LUAD cells inhibited proliferation, migration, and invasion, which suggest that GPPR115 was a tumor-promoting gene.

To further explore the molecular basis for the involvement of GPR115 in LUAD, we screened the significant DEGs based on high and low expression of GPR115 in TCGA-LUAD cohort. Excluding 530 down-regulated genes unrelated to LUAD prognosis, 378 up-regulated genes were included in WGCNA analysis. And the gray module showed the highest correlation with GPR115 expression, overall survival, tumor stage, tumor size and lymph node metastasis. Then GO analysis indicated GPR115-related genes of gray module were strongly associated with skin, epidermis cell and keratinocyte development, which was consistent with the higher GPR115 expression in human skin tissues (26). And KEGG pathway enrichment analysis illustrated that ECM-receptor interaction and Focal adhesion were interested with DEGs. The role of ECM and focal adhesion were proved in various tumors. ECM was abnormal expression in prostate cancer, gastric cancer and glioblastoma, and involved in tumor cell metastasis and infiltration (27–29). And the progression of epithelial mesenchymal transition (EMT) in colorectal cancer was activated by ECM (30). Focal adhesion kinase is a non-receptor tyrosine kinase that is involved in multiple signal transductions such as tumor proliferation, migration and vascular regeneration (31). In conclusion, these results determined the indispensable role of GPR115 in LUAD metastasis.

By calculated the correlation coefficient of GPR115 and ECM/Focal adhesion related genes, we found that LAMC2, the highest relevant gene, was remarkedly elevated in tumor tissues and positively associated with poor prognosis. LAMC2 is a subunit of the heterotrimeric glycoprotein laminin 332, which serves as a top mesenchymal marker (32). It has been reported that LAMC2 promotes cell invasion and migration by mediating EGFR activation and inducing the EMT process of CCA cells (33). Moon et al. also found that LAMC2 upregulates the mesenchymal marker -Vimentin expression in LUAD cells and enhances cell traction, proliferation and migration (34). For EMT transformation, on one hand, the decreased of epithelia marker N-cadherin inhibited cell adhesion. On the other hand, the epithelial cancer cells express mesenchymal proteins such as vimentin and N- cadherin, and become mesenchymal cells, ready for migration (35). Indeed, we found that siRNA-mediated downregulation of GPR115 greatly increased the expression of E-cadherin and decreased the expression of N-cadherin, and vimentin, which indicated that GPR115 may serve as a contributor in EMT. Therefore, we speculated that LAMC2 as an intermediate molecule of GPR115 regulated EMT progression, and inducing malignant phenotype of tumor. Fortunately, it was found that the high expression of GPR115 was accompanied by the increase of LAMC2 in the TCGA analysis. We have reason to believe that GPR115 cooperates with the high expression of LAMC2 to induce the mesenchymal changes of LUAD epithelial cells, thereby achieving the purpose of promoting metastasis.

In conclusion, this study was the first to determine GPR115 expression pattern in NSCLC, its value as a prognostic marker for LUAD patients, and its functional role as a tumor promoter. We also identified LAMC2 with significantly related expression to GPR115 that promotes tumor metastasis for LUAD. Collectively, these data suggest that GRP115 may play important roles in the development of LUAD and may have utility as diagnostic biomarkers and/or therapeutic targets. And GPR115 might activated tumor malignant progression by correlated with LAMC2 to enhanced EMT development. However, the limitations of this study were that it involved only in vitro study. And as a retrospective study, the results in this study are susceptible to sample size and selection bias, although we try to expand the sample size as much as possible. Next, we will try to construct a GPR115-deletion mouse model to verify that GPR115 regulates tumor proliferation and metastasis, which was important to further promote our results toward clinical applications.

## Data Availability Statement

The raw data supporting the conclusions of this article will be made available by the authors, without undue reservation.

## Ethics Statement

The studies involving human participants were reviewed and approved by the Human Research Ethics Committee of the Affiliated Hospital of Nantong University, Jiangsu, China. The patients/participants provided their written informed consent to participate in this study.

## Author Contributions

Guarantor of integrity of the entire study: LX. Study concepts and design: LX and XZ. Literature research and experimental execution: YW. Data analysis and statistical analysis: YW, MS, and NY. Manuscript editing: YW. Final approval of manuscript: All authors. All authors contributed to the article and approved the submitted version.

## Funding

This work was supported by the Jiangsu Provincial “13th Five-Year Plan” Key Science and Technology Talent Subsidy Project (ZDRCA2016051), the Jiangsu Provincial Medical Youth Talent (WQ2016077), the Nantong Science and Technology Bureau (MSI2018038), and the Nantong Municipal Science and Technology Plan (MS12018037).

## Conflict of Interest

The authors declare that the research was conducted in the absence of any commercial or financial relationships that could be construed as a potential conflict of interest.

## References

[1] Bray F Ferlay J Soerjomataram I Siegel RL Torre LA and Jemal A.: Global cancer statistics 2018: GLOBOCAN estimates of incidence and mortality worldwide for 36 cancers in 185 countries. CA Cancer J Clin (2018) 68(6):394–424. 10.3322/caac.21492 30207593

[2] Nadal E Massuti B Domine M Garcia-Campelo R Cobo M Felip E Immunotherapy with checkpoint inhibitors in non-small cell lung cancer: insights from long-term survivors. Cancer Immunol Immunother (2019) 68(3):341–52. 10.1007/s00262-019-02310-2 PMC11028247 30725206

[3] Chen Z Fillmore CM Hammerman PS Kim CF Wong KK Non-small-cell lung cancers: a heterogeneous set of diseases. Nat Rev Cancer (2014) 14(8):535–46. 10.1038/nrc3775 PMC5712844 25056707

[4] Iaboni M Russo V Fontanella R Roscigno G Fiore D Donnarumma E Aptamer-miRNA-212 Conjugate Sensitizes NSCLC Cells to TRAIL. Mol Ther Nucleic Acids (2016) 5:e289. 10.1038/mtna.2016.5 27111415 PMC5014461

[5] Chen K Liu H Liu Z Luo S Patz EF Jr. Moorman PG Genetic variants in RUNX3, AMD1 and MSRA in the methionine metabolic pathway and survival in nonsmall cell lung cancer patients. Int J Cancer (2019) 145(3):621–31. 10.1002/ijc.32128 PMC6828159 30650190

[6] Wu Y Xu M He R Xu K Ma Y ARHGAP6 regulates the proliferation, migration and invasion of lung cancer cells. Oncol Rep (2019) 41(4):2281–888. 10.3892/or.2019.7031 30816546

[7] Remon J Hendriks LE Cabrera C Reguart N Besse B Immunotherapy for oncogenic-driven advanced non-small cell lung cancers: Is the time ripe for a change? Cancer Treat Rev (2018) 71:47–58. 10.1016/j.ctrv.2018.10.006 30359792

[8] Almendro V Garcia-Recio S Gascon P Tyrosine kinase receptor transactivation associated to G protein-coupled receptors. Curr Drug Targets (2010) 11(9):1169–80. 10.2174/138945010792006807 20450475

[9] Ritter SL Hall RA Fine-tuning of GPCR activity by receptor-interacting proteins. Nat Rev Mol Cell Biol (2009) 10(12):819–30. 10.1038/nrm2803 PMC2825052 19935667

[10] Liu Y An S Ward R Yang Y Guo XX Li W G protein-coupled receptors as promising cancer targets. Cancer Lett (2016) 376(2):226–39. 10.1016/j.canlet.2016.03.031 27000991

[11] Hamann J Aust G Arac D Engel FB Formstone C Fredriksson R International Union of Basic and Clinical Pharmacology. XCIV. Adhesion G protein-coupled receptors. Pharmacol Rev (2015) 67(2):338–67. 10.1124/pr.114.009647 PMC4394687 25713288

[12] Zhang W Cui Q Qu W Ding X Jiang D Liu H TRIM58/cg26157385 methylation is associated with eight prognostic genes in lung squamous cell carcinoma. Oncol Rep (2018) 40(1):206–16. 10.3892/or.2018.6426 PMC6059744 29749538

[13] Chiba Y Yoshizaki K Saito K Ikeuchi T Iwamoto T Rhodes C G-protein coupled receptor Gpr115 (Adgrf4) is required for enamel mineralization mediated by ameloblasts. J Biol Chem (2020) jbc.RA120.014281. 10.1074/jbc.RA120.014281 PMC7650236 32868297

[14] Ozer B Sezerman U Analysis of the interplay between methylation and expression reveals its potential role in cancer aetiology. Funct Integr Genomics (2017) 17(1):53–68. 10.1007/s10142-016-0533-9 27819121

[15] Zeng Y Mayne N Yang CJ D’Amico TA Ng CSH Liu CC A Nomogram for Predicting Cancer-Specific Survival of TNM 8th Edition Stage I Non-small-cell Lung Cancer. Ann Surg Oncol (2019) 26(7):2053–62. 10.1245/s10434-019-07318-7 30900105

[16] Huang J Mei H Tang Z Li J Zhang X Lu Y Triple-amiRNA VEGFRs inhibition in pancreatic cancer improves the efficacy of chemotherapy through EMT regulation. J Control Release (2017) 245:1–14. 10.1016/j.jconrel.2016.11.024 27889393

[17] Pei G Chen L Zhang W WGCNA Application to Proteomic and Metabolomic Data Analysis. Methods Enzymol (2017) 585:135–58. 10.1016/bs.mie.2016.09.016 28109426

[18] Gao J Aksoy BA Dogrusoz U Dresdner G Gross B Sumer SO Integrative analysis of complex cancer genomics and clinical profiles using the cBioPortal. Sci Signal (2013) 6(269):pl1. 10.1126/scisignal.2004088 23550210 PMC4160307

[19] Steeg PS Tumor metastasis: mechanistic insights and clinical challenges. Nat Med (2006) 12(8):895–904. 10.1038/nm1469 16892035

[20] Krasnoperov VG Bittner MA Beavis R Kuang Y Salnikow KV Chepurny OG alpha-Latrotoxin stimulates exocytosis by the interaction with a neuronal G-protein-coupled receptor. Neuron (1997) 18(6):925–37. 10.1016/S0896-6273(00)80332-3 9208860

[21] Steinert M Wobus M Boltze C Schutz A Wahlbuhl M Hamann J Expression and regulation of CD97 in colorectal carcinoma cell lines and tumor tissues. Am J Pathol (2002) 161(5):1657–67. 10.1016/S0002-9440(10)64443-4 PMC1850798 12414513

[22] Davies B Baumann C Kirchhoff C Ivell R Nubbemeyer R Habenicht UF Targeted deletion of the epididymal receptor HE6 results in fluid dysregulation and male infertility. Mol Cell Biol (2004) 24(19):8642–8. 10.1128/MCB.24.19.8642-8648.2004 PMC516748 15367682

[23] Lin HH Faunce DE Stacey M Terajewicz A Nakamura T Zhang-Hoover J The macrophage F4/80 receptor is required for the induction of antigen-specific efferent regulatory T cells in peripheral tolerance. J Exp Med (2005) 201(10):1615–25. 10.1084/jem.20042307 PMC2212925 15883173

[24] Jeong SJ Luo R Li S Strokes N Piao X Characterization of G protein-coupled receptor 56 protein expression in the mouse developing neocortex. J Comp Neurol (2012) 520(13):2930–40. 10.1002/cne.23076 PMC3908671 22351047

[25] Ward Y Lake R Martin PL Killian K Salerno P Wang T CD97 amplifies LPA receptor signaling and promotes thyroid cancer progression in a mouse model. Oncogene (2013) 32(22):2726–38. 10.1038/onc.2012.301 PMC7561260 22797060

[26] Gerber PA Hevezi P Buhren BA Martinez C Schrumpf H Gasis M Systematic identification and characterization of novel human skin-associated genes encoding membrane and secreted proteins. PloS One (2013) 8(6):e63949. 10.1371/journal.pone.0063949 23840300 PMC3688712

[27] Andersen MK Rise K Giskeodegard GF Richardsen E Bertilsson H Storkersen O Integrative metabolic and transcriptomic profiling of prostate cancer tissue containing reactive stroma. Sci Rep (2018) 8(1):14269. 10.1038/s41598-018-32549-1 30250137 PMC6155140

[28] Yan P He Y Xie K Kong S Zhao W In silico analyses for potential key genes associated with gastric cancer. PeerJ (2018) 6:e6092. 10.7717/peerj.6092 30568862 PMC6287586

[29] Cui X Morales RT Qian W Wang H Gagner JP Dolgalev I Hacking macrophage-associated immunosuppression for regulating glioblastoma angiogenesis. Biomaterials (2018) 161:164–78. 10.1016/j.biomaterials.2018.01.053 PMC8059366 29421553

[30] Rahbari NN Kedrin D Incio J Liu H Ho WW Nia HT Anti-VEGF therapy induces ECM remodeling and mechanical barriers to therapy in colorectal cancer liver metastases. Sci Transl Med (2016) 8(360):360ra135. 10.1126/scitranslmed.aaf5219 PMC5457741 27733559

[31] Rustad KC Wong VW Gurtner GC The role of focal adhesion complexes in fibroblast mechanotransduction during scar formation. Differentiation (2013) 86(3):87–91. 10.1016/j.diff.2013.02.003 23623400

[32] Parikh AS Puram SV Faquin WC Richmon JD Emerick KS Deschler DG Immunohistochemical quantification of partial-EMT in oral cavity squamous cell carcinoma primary tumors is associated with nodal metastasis. Oral Oncol (2019) 99:104458. 10.1016/j.oraloncology.2019.104458 31704557 PMC7382966

[33] Pei YF Liu J Cheng J Wu WD Liu XQ Silencing of LAMC2 Reverses Epithelial-Mesenchymal Transition and Inhibits Angiogenesis in Cholangiocarcinoma via Inactivation of the Epidermal Growth Factor Receptor Signaling Pathway. Am J Pathol (2019) 189(8):1637–53. 10.1016/j.ajpath.2019.03.012 31345467

[34] Moon YW Rao G Kim JJ Shim HS Park KS An SS LAMC2 enhances the metastatic potential of lung adenocarcinoma. Cell Death Differ (2015) 22(8):1341–52. 10.1038/cdd.2014.228 PMC4495359 25591736

[35] Tsoukalas N Aravantinou-Fatorou E Tolia M Giaginis C Galanopoulos M Kiakou M Epithelial-Mesenchymal Transition in Non Small-cell Lung Cancer. Anticancer Res (2017) 37(4):1773–8. 10.21873/anticanres.11510 28373440

